# Regenerative Potential of Hydrogels for Intracerebral Hemorrhage: Lessons from Ischemic Stroke and Traumatic Brain Injury Research

**DOI:** 10.1002/adhm.202100455

**Published:** 2021-07-01

**Authors:** Josephine M. Thomas, Irene Louca, Faye Bolan, Oana‐Roxana Sava, Stuart M. Allan, Catherine B. Lawrence, Emmanuel Pinteaux

**Affiliations:** ^1^ Geoffrey Jefferson Brain Research Centre The Manchester Academic Health Science Centre Northern Care Alliance NHS Group The University of Manchester Manchester M13 9PT UK; ^2^ Division of Neuroscience and Experimental Psychology Faculty of Biology Medicine and Health The University of Manchester Manchester M13 9PT UK

**Keywords:** acute brain injury, hydrogels, intracerebral hemorrhage, regenerative medicine, stroke

## Abstract

Intracerebral hemorrhage (ICH) is a deadly and debilitating type of stroke, caused by the rupture of cerebral blood vessels. To date, there are no restorative interventions approved for use in ICH patients, highlighting a critical unmet need. ICH shares some pathological features with other acute brain injuries such as ischemic stroke (IS) and traumatic brain injury (TBI), including the loss of brain tissue, disruption of the blood–brain barrier, and activation of a potent inflammatory response. New biomaterials such as hydrogels have been recently investigated for their therapeutic benefit in both experimental IS and TBI, owing to their provision of architectural support for damaged brain tissue and ability to deliver cellular and molecular therapies. Conversely, research on the use of hydrogels for ICH therapy is still in its infancy, with very few published reports investigating their therapeutic potential. Here, the published use of hydrogels in experimental ICH is commented upon and how approaches reported in the IS and TBI fields may be applied to ICH research to inform the design of future therapies is described. Unique aspects of ICH that are distinct from IS and TBI that should be considered when translating biomaterial‐based therapies between disease models are also highlighted.

## Graphical abstract

This work reviews discoveries reported in ischemic stroke (IS) and traumatic brain injury (TBI), and how these can be exploited for the translational application of hydrogels alone or in combination therapies (stem cells) in intracerebral hemorrhage (ICH). The review highlights hallmarks of ICH that are distinct from IS and TBI that need considering when translating biomaterial‐based therapies between disease models.

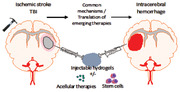

## 1 Introduction

Injury of brain tissue caused by internal or external factors is a common feature of stroke and head trauma. Brain injury can be caused by blunt force, tissue ischemia, or hemorrhage, as observed in traumatic brain injury (TBI), ischemic stroke (IS), and intracerebral hemorrhage (ICH), respectively.^[^ ^1^ ^]^ Despite differences in disease mechanisms, clinical impact, and predicted outcomes, all three conditions share common pathological features. These shared characteristics can be exploited when investigating the translational potential of tissue engineering therapeutics for new clinical applications. The use of hydrogels as bioactive scaffolds or therapeutic delivery vehicles has been widely investigated in the field of IS and TBI but is still in relative infancy for use in ICH. This review is not intended to provide a comprehensive overview of the use of hydrogels in acute brain injury, rather to briefly discuss promising approaches that could be applied to the nascent field of biomaterials in ICH. Recently, Love et al. raised important questions concerning the use of biomaterials for stroke therapy.^[^ ^2^ ^]^ Here, we expand upon this by identifying key themes from the extensive IS and TBI literature that can inform regenerative medicine approaches specifically for ICH. We briefly cover the commonalities of acute brain injury and the unique considerations for ICH, discuss the experimental approaches used to date, and consider how methods from IS and TBI studies may present opportunities to advance this emerging field of ICH research.

## 2 Common Mechanisms of Acute Brain Injury

Acute brain injury alters the physiological architecture and function of the brain tissue, resulting in a pathology with clinical symptoms. The common hallmarks characterizing disease progression during brain injury include the acute loss of brain tissue and disruption of the blood–brain barrier (BBB).^[^ ^1^, ^3^ ^]^ Loss of neural and vascular brain tissue can arise as a result of tissue ischemia as observed in IS, following mechanical damage downstream of hematoma and edema formation in ICH, or due to external forces to the skull after a contusion in TBI (**Table** **1**).^[^ ^4^, ^5^ ^]^

**Table 1:** Differences in acute brain injury progression with possible impacts on hydrogel therapy design

	IS (blockage of cerebral blood vessels)	TBI (trauma to the skull)	ICH (rupture of cerebral blood vessels)	Considerations for hydrogel therapy
Major risk factors	Age Hypertension Hypercholesterolemia Smoking Cardiovascular disease	N/A (caused by trauma)	Age Hypertension Hypercholesterolemia Smoking Cardiovascular disease Cerebral amyloid angiopathy	Therapies may need to be personalized based on underlying health conditions affecting the brain microarchitecture
Injury location	Cortical and subcortical	Predominantly cortical	Cortical and subcortical, subcortical more prevalent	Hydrogel therapies should be tailored for brain regions with different cell types and physical environments
Blood toxicity	N/A unless hemorrhagic transformation	+	+++	Hydrogels may need to be tailored to reduce blood toxicity for ICH, e.g., by inclusion of iron chelators
Elevated ICP	+	+	++	Volume of hydrogel administered and the effect on ICP must be considered
Recruitment of immune cells and inflammatory response	Acute phase: reactive astrocytes, mixed microglial phenotype, infiltration of inflammatory monocytes Chronic phase: shift to M1 (proinflammatory) microglia, glial scar formation	Acute phase: reactive astrocytes, mixed microglial phenotype, infiltration of inflammatory monocytes Chronic phase: shift to M1 microglia, glial scar formation	Acute phase: reactive astrocytes, mostly M1 microglia, infiltration of inflammatory monocytes Chronic phase: shift to M2 (reparative) microglia, glial scar formation	Interactions of resident and infiltrating cells with hydrogel therapies should be considered, especially with regards to timing of administration
Cavity formation at lesion site	+	+	+/++ Depending on hematoma aspiration	Volumes of hydrogel injection should be compatible with cavity volume

Following initial tissue injury, secondary damage is caused by a potent inflammatory response. Activation of the immune system and a local inflammatory reaction is observed in all three types of brain injury.^[^ ^6^, ^7^, ^8^ ^]^ The inflammatory cascade is characterized by activation of tissue‐resident microglia and astrocytes, and infiltration of peripheral immune cells such as neutrophils, monocytes, and macrophages.^[^ ^9^, ^10^ ^]^ Reactive astrocytes and microglia are responsible for the formation of a glial scar, which forms a border around the injury site isolating it from healthy tissue to prevent further damage. BBB integrity and water transport homeostasis are markedly affected upon astrogliosis and microgliosis in the glial scar, which trigger neurodegenerative as well as neuroprotective responses in these pathological conditions.^[^ ^11^ ^]^

Acute brain injury progression is further promoted by inflammatory cytokine cascades that exacerbate tissue loss and functional impairment, resulting in sensorimotor and cognitive deficits.^[^ ^12^, ^13^, ^14^ ^]^ Patients may improve with rehabilitation and supportive interventions; however, the brain is unable to fully repair and recover to its preinjury state, leaving survivors of brain injury with significant disabilities. This is a result of the poor regenerative capacity of the brain, despite the presence of endogenous repair mechanisms such as neurogenesis where tissue‐resident neural stem cells (NSCs) are recruited from the subventricular and subgranular neurogenic zones.^[^ ^15^, ^16^ ^]^ However, NSCs are often prevented from reaching the injury site by the impenetrable glial scar or are insufficient in number to repopulate the lesion for adequate recovery.^[^ ^17^ ^]^ Additionally, neovascularization and blood vessel remodeling occur in an attempt to restore the broken‐down BBB and re‐establish blood flow to support the viability of the newly repaired neural tissue.^[^ ^1^ ^]^

## 3 Unique Pathophysiological Features of ICH

Despite the numerous common features of acute brain injury, there are some unique pathophysiological features of ICH, which require consideration when designing an intracerebral hydrogel treatment approach (Table 1). For instance, although edema is common to all acute brain injuries and causes an acute and often detrimental increase in intracranial pressure (ICP), the additional presence of a hematoma in the parenchyma in ICH can lead to dangerously sharp rises in ICP.^[^ ^18^ ^]^ The hematoma occupies space in the brain and elicits mass effects by compressing adjacent tissues which can cause neurovascular unit dysfunction including membrane depolarization and BBB disruption.^[^ ^19^, ^20^ ^]^ The combined effect of brain swelling and mass effects on ICP are prognostic of poorer outcomes and increased risk of mortality after ICH.^[^ ^19^, ^21^ ^]^

Another distinct feature of ICH is the exposure of neural tissue to blood and its degradation products. Thrombin and hemoglobin breakdown products such as heme and iron are released into the parenchyma upon hemolysis and trigger a cascade of secondary injury characterized by an exacerbated inflammatory response, oxidative stress, excitotoxicity, and cell death.^[^ ^22^, ^23^ ^]^ For instance, iron is thought to contribute to oxidative stress through the generation of free radicals,^[^ ^22^ ^]^ while thrombin is known to cause BBB disruption and thus worsening of edema.^[^ ^23^ ^]^ The unique features of ICH have been targeted in numerous preclinical and clinical interventions to date, with limited success.

## 4 Clinical Interventions in ICH

Clinical and preclinical trials have attempted to address the unmet clinical need in ICH. Nevertheless, ICH remains the deadliest subtype of stroke with case fatality reaching 40% after 1 month and 54% after a year.^[^ ^20^ ^]^ Therapeutic strategies investigated include pharmacological lowering of blood pressure to prevent hematoma expansion^[^ ^24^, ^25^ ^]^ and administration of iron chelators to reduce iron‐induced toxicity.^[^ ^26^, ^27^ ^]^ Despite promising preclinical results, to date, no pharmacological therapy has led to any clinical benefit after ICH when compared to standard medical care.^[^ ^28^ ^]^

A widely tested nonpharmacological therapeutic approach in ICH is surgical evacuation of the hematoma, either by craniotomy or minimally invasive surgery. Numerous active trials including the DIST (Dutch Intracerebral Hemorrhage Trial, NCT03608423), ENRICH (Early MiNimally invasive Removal of IntraCerebral Hemorrhage, NCT02880878), and MIND (Artemis in the Removal of Intracerebral Hemorrhage, NCT03342664) are investigating the efficacy of minimally invasive hematoma evacuation using specialized surgical technologies, with results still awaited. Another trial exploring minimally invasive surgical evacuation after ICH, the MISTIE III trial (Minimally Invasive Surgery with Thrombolysis for Intracerebral Hemorrhage Evacuation), was completed in 2019 and reported a neutral result of the intervention on primary functional outcomes.^[^ ^29^ ^]^ However, analysis of secondary outcomes suggested a modest improvement in mortality rates among treated patients and the trial demonstrated the safety of the surgical approach.^[^ ^29^ ^]^ This may open the possibility of a dual‐therapeutic approach combining surgical hematoma aspiration with the delivery of a therapeutic agent to provide enhanced recovery.

## 5 Emerging Preclinical Therapies for ICH

Preclinical ICH studies have investigated a range of novel therapeutic approaches, including transplantation of cells and administration of exogenous growth factors or drugs. Many studies have investigated the potential of mesenchymal stem cells (MSCs) as agents to elicit protection and repair in ICH models, frequently demonstrating improved functional recovery.^[^ ^30^ ^]^ NSCs^[^ ^31^ ^]^ and endothelial progenitor cells (EPCs),^[^ ^32^ ^]^ administered via direct intracerebral implantation or intravenous infusion, have also been studied as potential treatments in ICH due to their neurogenic and angiogenic properties, respectively. It has been proposed that the reparative effects observed following stem cell administration may be due to their paracrine actions, as evidence shows poor survival^[^ ^33^ ^]^ and engraftment in the brain,^[^ ^34^ ^]^ and marked accumulation in the lungs following systemic administration.^[^ ^35^ ^]^

Recent studies have explored the use of acellular therapies in ICH models, demonstrating positive impacts on functional recovery. These include the administration of conditioned medium, which contains factors released from cultured cells,^[^ ^36^ ^]^ and nanoscale membrane‐bound extracellular vesicles that carry cell‐derived cargos to near and distant sites.^[^ ^37^, ^38^ ^]^ Other acellular therapies being investigated for ICH include the delivery of growth factors such as fibroblast growth factor (FGF)^[^ ^39^ ^]^ and brain‐derived neurotrophic factor (BDNF),^[^ ^40^ ^]^ though persistence of these factors in the brain is limited, with exogenous growth factors diffusing into the cerebrospinal fluid within 12 h of injection. Pharmaceutical agents such as curcumin target brain edema by reducing BBB disruption following ICH,^[^ ^41^ ^]^ with other agents including dexamethasone, prostaglandin E2, and melatonin also being explored.^[^ ^42^ ^]^

While these novel therapeutic approaches have proven beneficial in animal models of ICH, the main barriers to clinical translation are the short retention time of cells and drugs in the brain and limited contact with perihematomal tissue.^[^ ^30^ ^]^ These issues give rise to the potential need for repeated administration over long‐term recovery periods or incorporation in material‐based sustained release systems to improve bioavailability.

## 6 Hydrogels for Acute Brain Injury Repair

To address current limitations in cell and drug delivery, soft biomaterials such as hydrogels have been considered for implantation at the ICH lesion site. Hydrogels are highly tunable materials of different origins – natural, such as collagen^[^ ^43^ ^]^ or hyaluronic acid (HA)^[^ ^44^ ^]^ hydrogels, and synthetic, such as self‐assembling peptide hydrogels (SAPHs).^[^ ^45^, ^46^ ^]^ Hydrogels are ideal candidates for tissue regeneration as they can be engineered to mimic the native extracellular matrix (ECM) and provide structural support for repair processes.^[^ ^47^ ^]^ They can also be functionalized with cell adhesion motifs required for the attachment of transplanted cells or endogenous brain cells recruited for regeneration.^[^ ^46^ ^]^

The use of hydrogels for repair following IS and TBI has been extensively reviewed elsewhere and will not be covered in detail here.^[^ ^48^, ^49^, ^50^ ^]^ In general, for neural tissue applications, hydrogels should be biodegradable and tailored to match the brain stiffness.^[^ ^51^ ^]^

The use of temporary scaffolds is desirable to initiate and support de novo tissue formation, however gradual degradation of the hydrogel may lead to altered mechanical properties such as a reduction in hydrogel stiffness. In vitro evidence suggests that stiffer hydrogels favor the differentiation of neural progenitors toward astrocytic lineages compared to softer substrates, which promote neuronal differentiation.^[^ ^52^ ^]^ The in vivo implantation of hydrogels with stiffness exceeding that of normal brain tissue can result in an exacerbated glial scar.^[^ ^53^ ^]^ However, very soft gels with fast degradation rates may not persist in situ for sufficient time to allow brain tissue regeneration. Therefore, hydrogel stiffness and degradation profile should be carefully considered to achieve a balance between supporting regeneration and enabling rapid clearance.

For minimally invasive administration, these hydrated materials need to be injectable and ideally gel in situ, in order to fill irregularly shaped cavities resulting from injuries including ICH.^[^ ^45^, ^47^, ^54^, ^55^, ^56^ ^]^ Engineered hydrogels can release encapsulated therapeutics in a sustained manner, and therefore prolong drug presence around the target tissue, reducing the dose needed and avoiding systemic side effects.^[^ ^55^, ^57^ ^]^ Hydrogels can also be used to deliver cells into the ICH cavity,^[^ ^58^ ^]^ enhancing cell survival by preventing mechanical damage to cells upon injection^[^ ^59^ ^]^ and providing a permissive microenvironment for cell engraftment.^[^ ^60^ ^]^

## 7 Considerations for Hydrogel Application in ICH

To the best of our knowledge, to date, only four groups have investigated hydrogel‐based therapies in animal models of ICH, demonstrating positive impacts on functional and histological outcomes following intracerebral hydrogel injection (**Table** **2**). Consistent with the general ICH literature, the collagenase model is the most widely used for these studies, with rats being the predominant species, likely due to the ability to inject larger gel volumes into the ICH cavity. Several of these studies aspirate the hematoma within hours of ICH induction, filling the resulting cavity with hydrogel, while others administer the hydrogel acutely (within 6 h) or chronically (after 14 days) after ICH without aspiration. Composition of the hydrogel itself is varied; some groups employ natural gels, composed of gelatin or keratin, while others use SAPHs that are based on RADA (arginine, alanine, and aspartate) amino acid repeats. Some of these studies have incorporated previously tested pharmacological agents in the gels, including deferoxamine mesylate (DFO) and minocyline hydrochloride (MH), which have shown no benefit in clinical trial when injected alone, suggesting that the synergistic effect of the drug–hydrogel combination may benefit recovery. Given the unique pathological features of ICH, it is critical to consider all aspects of in vivo study design, from the time of administration to the outcomes measured. These factors will now be discussed, with considerations as to how research in IS and TBI may be translatable to ICH studies.

**Table 2:** Published use of hydrogel therapies in ICH models. Outcomes refer to quantified histological or behavioral data of hydrogel group(s) which are significantly altered compared with control groups. Abbreviations: bFGF, basic fibroblast growth factor; BMSCs, bone‐marrow‐derived mesenchymal stem cells; BrdU, bromodeoxyuridine; DFO, deferoxamine mesylate; EGF, epidermal growth factor; Iba‐1, ionized calcium‐binding adapter molecule 1; GFAP, glial fibrillary acidic protein; ICH, intracerebral hemorrhage; MH, minocyline hydrochloride; mNSS, modified neurological severity score; MPO, myeloperoxidase; NeuN, hexaribonucleotide binding protein‐3; nNOS, neuronal nitric oxide synthase; NPCs, neural progenitor cells; TNF‐α, tumour necrosis factor alpha; TUNEL, terminal deoxynucleotidyl transferase; dUTP, nick end labeling

Study	Model	Time of administration	Hydrogel base	Encapsulated agents	Outcomes
Xu et al. 2020^[^ ^92^ ^]^	Collagenase, mouse	3 days post‐ICH	Gelatin	n/a	↑ Neuronal density (NeuN) ↓ Activated microglia (Iba‐1) and astrocytes (GFAP), release of interleukin‐1*β* and TNF‐*α* ↑ Functional recovery assessed by neuroscore
Lim et al. 2020^[^ ^56^ ^]^	Collagenase, rat	14 days post‐ICH	Gelatin and hydrophenylproprionic acid	EGF	↑ NPCs (Nestin+) migrating into cavity ↑ Functional recovery assessed by mNSS, forelimb placing, and corner test scores
Gong et al. 2020^a,b)^ ^[^ ^58^ ^]^	FeCl_2_, rat	0 h (ICH surgery)	Keratin‐based core–shell hydrogel	EGF, bFGF, MH, BMSCs	↓ Iron staining, brain atrophy, and brain water content ↑ Proliferation (BrdU) at 14 days ↑ Functional recovery assessed by forelimb placing and corner test scores
Zhu et al. 2019^a,b)^ ^[^ ^55^ ^]^	Autologous blood, rat	6 h post‐ICH	Keratin and *N*‐isopropyl acrylamide	DFO	↓ Iron staining, nonheme iron content, and brain water content ↑ Functional recovery assessed by forelimb placing and corner test scores
Luo et al. 2017^a,b)^ ^[^ ^57^ ^]^	Autologous blood + aspiration at 4 h, rat	4 h post‐ICH	Keratose	MH	↓ Iron staining, iron content, and brain water content ↑ Functional recovery assessed by forelimb placing and corner turn test
Zhang et al. 2016^a,b)^ ^[^ ^46^ ^]^	Collagenase + aspiration at 3.5 h, mouse	3.5 h post‐ICH	RADA16–RGD/RADA16–IKVAV mix^c)^	n/a	↓ Hematoma volume, microglia (Iba‐1), infiltrating macrophages (CD11b), C‐jun, and apoptosis (TUNEL) ↑ Neurons (NeuN) and neuron survival (nNOS) ↑ Functional recovery assessed by rotarod, grip strength, and gait analysis
Sang et al. 2015^a,b)^ ^[^ ^45^ ^]^	Collagenase + aspiration at 3.5 h, rat	3.5 h post‐ICH	RADA16‐I^c)^	n/a	↓ Hematoma volume and brain water content ↓ Neutrophils (MPO+), microglia/macrophages (CD68), and apoptosis (TUNEL) in perihematomal area
Sun et al. 2016^a,b)^ ^[^ ^91^ ^]^	Collagenase + aspiration at 3.5 h, mouse	3.5 h post‐ICH	RADA 16‐I or *RADA 16–IKVAV/RADA 16–RGD^c)^	n/a	Unclear outcomes of hydrogel injection

^a,b)^ Studies by the same group

^c)^ Amino acid sequences for peptide hydrogels.

## 8 Study Design: Time of Administration

Therapeutic interventions for brain injuries typically target two distinct therapeutic windows. Neuroprotective therapies are given early (within hours) and aim to minimize tissue damage and functional impairment, while regenerative therapies are typically delayed (days to weeks) and aim to promote tissue recovery and restore physiological tissue architecture and function.^[^ ^54^ ^]^ For the purpose of this article, we define administration intervals as follows: acute as within 24 h, subacute as between 2 and 5 days, and chronic as beyond 5 days post‐ICH.

The target and mechanism of action of new treatments must be considered when determining the: i) timing and volume of hydrogel administration, ii) timepoints for assessment, and iii) primary outcome measures, as all will have a decisive role on the conclusions that can be made from the study. The time of administration is critical for the efficacy of the treatment. For example, administration of hydrogel therapies targeting the detrimental inflammatory response may be more appropriate during the acute and subacute stages of brain injury as tissue damage and inflammation are prominent shortly following tissue insult.^[^ ^61^, ^62^ ^]^ Conversely, transplantation of cell‐laden hydrogels for tissue repair in an environment rich in inflammatory cytokines, necrotic tissue, blood‐by‐products, and degrading enzymes can compromise the cells’ potential for integration and differentiation into the local tissue.^[^ ^62^ ^]^

The small number of preclinical studies in ICH (Table 2) are primarily focused on acute administration of hydrogels with six out of eight studies administering the hydrogel within 6 h of ICH induction. Early hydrogel transplantation is preceded by hematoma evacuation in four studies, while only one targets the chronic window for tissue repair by transplanting hydrogels in the ICH cavity 14 days after hemorrhage induction (Table 2). A similar trend is observed in the literature of hydrogel studies in TBI where many studies target the acute neuroinflammation phase of TBI with hydrogels being administered immediately after TBI induction or within the first hours following injury. A small number of studies administered hydrogels at 7 days post‐TBI.^[^ ^63^, ^64^, ^65^ ^]^ A systematic review and meta‐analysis conducted by our group on in vivo studies using biomaterials in experimental IS revealed that hydrogels are predominantly transplanted during the chronic reparative phase. Most studies administered hydrogels days or weeks after stroke, while few studies investigated hydrogel administration earlier than 24 h post‐IS.^[^ ^66^ ^]^ The difference in approaches for hydrogel treatment in IS versus ICH and TBI is noteworthy and future studies in ICH should consider delayed treatment paradigms.

## 9 Study Design: Combination of Hydrogels with Therapeutics

In ICH preclinical studies, hydrogels have been used as scaffolds for regeneration^[^ ^45^, ^46^ ^]^ or as delivery systems for therapeutics.^[^ ^55^, ^56^, ^57^, ^58^ ^]^ Approaches targeting unique aspects of ICH include the combination of hydrogels with previously tested drugs such as DFO^[^ ^55^ ^]^ and MH,^[^ ^57^ ^]^ which were delivered to the lesion for sustained action within a keratin‐based hydrogel. DFO was more effective in reducing iron deposition and brain edema when released from a hydrogel compared to DFO or hydrogel alone.^[^ ^55^ ^]^ Growth factors such as epidermal growth factor (EGF)^[^ ^56^ ^]^ and its combination with basic FGF (bFGF)^[^ ^58^ ^]^ have also been delivered using gelatin‐ and keratin‐based hydrogels, respectively. All these approaches were combined by Gong et al., who designed a core–shell hydrogel system containing MH and poly(lactic‐*co*‐glycolic acid) nanoparticles loaded with EGF and bFGF for bone‐marrow‐derived MSC delivery into an ICH model. The core–shell system improved the viability of transplanted cells and accelerated neurological recovery.^[^ ^58^ ^]^

Hydrogels have been extensively explored in IS and TBI studies as tools for delivering regenerative drugs, growth factors, or cells. Notable examples include slowly released BDNF from a HA hydrogel which reduced lesion volumes, improved performance in motor function tests at nine weeks post‐IS, and promoted axonal sprouting and neuroblast migration, when compared with bolus BDNF or hydrogel alone.^[^ ^67^ ^]^ Additionally, chondroitinase ABC stabilized in HA–methylcellulose blended hydrogels attenuated the glial scar in an IS model by reducing chondroitin sulfate proteoglycan levels at 28 days after injury.^[^ ^68^ ^]^ NSCs and EPCs transplanted either alone or together within a SAPH, 7 days post‐TBI, reduced astrogliosis at the lesion site compared to cells alone.^[^ ^69^ ^]^ Cotransplants of NSCs and adipose‐derived stem cells in the same hydrogel reduced brain lesion volumes, attenuated inflammation and glial scar formation, and improved motor function following TBI, compared to gel alone groups.^[^ ^70^ ^]^ Therapeutic combinations that show potential in IS and TBI may also prove beneficial in the context of ICH, given the shared disease mechanisms and regenerative processes involved in acute brain injuries. Additionally, multimodal therapies are yet to be explored in the context of ICH, presenting an opportunity to investigate cell‐laden hydrogel therapies. Transplantation of MSCs and NSCs in hydrogels has been proven beneficial in preclinical studies of IS and TBI, thus may also hold promise in aiding repair and regeneration post‐ICH.

## 10 Study Design: Assessing Regeneration

Endogenous repair mechanisms, such as neurogenesis and angiogenesis, are important hallmarks of tissue repair for gauging therapeutic efficacy of hydrogel interventions as both contribute to functional recovery after acute brain injury.^[^ ^71^, ^72^ ^]^ While tissue regeneration is associated with functional recovery after acute brain injury, the direct relationship between the two has not yet been fully elucidated. The assessment of functional recovery in ICH using a variety of behavioral tests has been extensively addressed and published previously.^[^ ^73^, ^74^, ^75^, ^76^ ^]^

At the tissue level, following an insult to the brain, resident neural stem or progenitor cells (NSPCs) are recruited from the neurogenic niches to the site of injury where they differentiate into neurons and glial cells in order to compensate for the lost cells.^[^ ^77^, ^78^ ^]^ For example, in IS, injection of a HA‐based hydrogel alone led to increased NSPC proliferation in the neurogenic niche of the brain, which were then shown to migrate into the core of the stroke lesion.^[^ ^79^ ^]^ To stimulate endogenous regenerative responses in TBI, a Matrigel scaffold with a gradient of semaphorin 3A was delivered, which led to an increase in migrating neuroblasts and NSPCs within the lesion compared to TBI alone.^[^ ^80^ ^]^ Although not as well characterized, a similar neurogenic response occurs following ICH.^[^ ^81^, ^82^, ^83^ ^]^ To further stimulate this response in experimental ICH, Lim et al. combined a gelatin‐based hydrogel with EGF and demonstrated that NSPCs could migrate into the hydrogel scaffold and differentiate into multiple neuronal lineages.^[^ ^56^ ^]^

Restorative neurogenesis is known to be coupled with simultaneous angiogenic processes.^[^ ^71^, ^72^ ^]^ NSPCs residing in the neurogenic niches are closely associated with the vasculature. Blood vessel remodeling via angiogenic and vasculogenic mechanisms facilitates neurogenesis and synaptogenesis as NSPCs have been demonstrated to rely on the vasculature for migration to the injury site.^[^ ^84^, ^85^, ^86^ ^]^ After ischemic injury, angiogenesis begins 4 to 7 days after onset, mainly at the border of the ischemic core.^[^ ^87^ ^]^ However, the process of neurogenesis does not start until around 2 weeks after stroke. It is thought that post‐ischemic angiogenesis may have a role in axonal outgrowth through vascular endothelial growth factor (VEGF) and laminin/*β*1‐integrin signaling. Endothelial cells orchestrate vascular remodeling and organize into new blood vessels to repair the damaged vascular network and re‐establish blood flow to the site of injury. New blood vessels formed early after stroke are also thought to increase the proliferation of NSPCs and support their recruitment to the site of injury, by providing a scaffold for migration as well as supplying oxygen and nutrients. Finally, tissue oxygenation in the ischemic core triggered by angiogenesis is thought to increase the differentiation of recruited NSPCs into mature neuronal cell types.^[^ ^88^ ^]^ These processes are thought to contribute to functional recovery.

The decoupling of these mechanisms is a common feature of acute brain injury and therefore therapeutic strategies targeting these reparative processes may hold promise.^[^ ^71^, ^72^ ^]^ For instance, delivery of a HA hydrogel with encapsulated VEGF into the cavity resulted in de novo blood vessel formation in both IS and TBI,^[^ ^44^, ^89^ ^]^ and the elaboration of functional axonal networks along the novel vasculature in regions of previously lost tissue in IS.^[^ ^44^ ^]^ Additionally, injection of the SAPH “SLanc” after TBI caused a significant upregulation of canonical markers of angiogenesis, including von Willebrand factor and VEGF receptor 2 compared with TBI control animals.^[^ ^90^ ^]^

To date, studies testing hydrogels in ICH have assessed tissue responses by observing changes in mature neural markers,^[^ ^46^ ^]^ by exploring immune cell infiltration^[^ ^45^, ^46^, ^56^ ^]^ or by assessing gross morphological changes such as ventricular enlargement and cavity volume.^[^ ^45^, ^46^ ^]^ While global analysis of the effect of hydrogel injection on the surrounding tissue is important, future work exploring the effect on endogenous repair mechanisms, for instance, on the recruitment of NSPCs and the expression of key angiogenic markers, will be of great value to the ICH field. However, it is worth noting that while hydrogels for neural repair are often designed to initiate neuronal cell differentiation, angiogenesis precedes and facilitates subsequent neurogenesis and is likely to occur under different conditions. In addition, hydrogels will undergo complex dynamic remodeling after in vivo implantation. Therefore, it is challenging to determine the exact hydrogel mechanical properties needed for distinct but interdependent repair processes during the course of regeneration.

## 11 Discussion of Unique Considerations for ICH

### 11.1 Hydrogel Properties

When designing hydrogels for ICH applications, many of the desirable properties for application in other acute brain injuries also apply. For instance, the candidate hydrogel should: i) be injectable and be able to gel in situ for minimally invasive administration, ii) have mechanical properties comparable to brain tissue, iii) be biocompatible and biodegradable to support de novo tissue formation, and iv) be made of an appropriate material with a porous structure that allows host and transplanted cell infiltration, adhesion, and survival, as well as circulation of metabolites. These key features have been largely addressed in hydrogel studies in ICH so far (Table 2).

Hydrogel properties are largely dependent on the material used for hydrogel formulation. The majority of studies in ICH use protein or peptide‐based hydrogels^[^ ^45^, ^46^, ^91^ ^]^ due to their favorable properties, facilitating in vivo degradation or gelation either via physical (SAPHs^[^ ^45^, ^46^, ^91^ ^]^) or chemical cross‐linking reactions (gelatin‐based hydrogels^[^ ^56^, ^92^ ^]^). Hydrogel functionalization has also been explored to enhance cell adhesion; studies using synthetic SAPHs have reported increased cell adhesion to the hydrogel after incorporation of ECM‐based adhesion motifs such as IKVAV (laminin) or RGD (fibronectin) peptides.^[^ ^46^, ^91^ ^]^ Hydrogel functionalization may also improve the survival of transplanted cells and host tissue and modulate the inflammatory response, leading to improved functional recovery.^[^ ^46^ ^]^ However, it is worth noting that natural hydrogels such as collagen and gelatin inherently contain endogenous cell adhesion motifs, thus, further functionalization may not be required.

Tuning the mechanical characteristics of the hydrogel such as stiffness and porosity is often necessary to ensure an appropriate tissue response and prevent damage to healthy tissue. For example, four studies evaluating hydrogels for ICH reported hydrogel stiffness in the range of normal brain tissue (10–1000 Pa).^[^ ^56^, ^58^, ^91^, ^92^ ^]^ The degree of scaffold porosity is also critical for facilitating cell invasion and circulation of nutrients and metabolites in the transplanted hydrogel. Several ICH papers assessing hydrogel porosity by scanning electron microscopy^[^ ^55^, ^57^, ^58^, ^92^ ^]^ reported the presence of interconnected network of pores with sizes between 20 and 100 µm, which falls within the range of mammalian cell diameter to facilitate cell infiltration. Conversely, hydrogels with smaller pore sizes, as reported for RADA‐I‐based SAPH (5–200 nm), may preclude or hinder cell infiltration.^[^ ^93^ ^]^ Hydrogel injectability is another desirable quality for transplantation and the majority of published studies using hydrogels in ICH use injectable hydrogels that gel in situ. Injectable hydrogels permit minimally invasive administration and allow gels to conform to the hematoma cavity space, minimizing pressure on surrounding brain structures and healthy tissue.

In addition to physical characteristics, biological properties of hydrogels such as biocompatibility and the ability to interact with the surrounding tissue can also be tailored for in vivo applications. Hydrogels administered after ICH were shown to be well tolerated by the host tissue as no studies reported an increase in inflammation, cell apoptosis, or scarring. For instance, SAPHs led to decreased cell apoptosis and reduced infiltration of inflammatory cells after ICH,^[^ ^45^, ^46^ ^]^ while administration of a gelatin hydrogel led to lower activated immune cell infiltration when compared to the vehicle group.^[^ ^92^ ^]^ Following administration into the brain, hydrogel interaction with transplanted and endogenous cells is critical for regeneration and functional recovery. In ICH, an EGF‐containing hydrogel was shown to improve NSPC recruitment compared to bolus EGF^[^ ^56^ ^]^ and administration of a RADA‐based SAPH decreased microglial and macrophage infiltration,^[^ ^46^ ^]^ while administration of a drug‐containing hydrogel led to improved graft BMSC viability and improved host neuronal cell survival.^[^ ^58^ ^]^ Finally, examination of material interaction with the hematoma revealed that the hydrogel may prevent the formation of a visible hemosiderin scar, which is a common feature of ICH pathology.^[^ ^92^ ^]^ Hydrogels such as RADA‐based hydrogels may also reduce the hematoma volume, revealing possible hemostatic properties.^[^ ^46^ ^]^

Overall, hydrogel studies in ICH models to date have considered the impact of hydrogel properties on a variety of functional and histological outcomes. However, given the limited number of published studies and groups investigating hydrogels for ICH, identifying an “ideal” hydrogel candidate for a regenerative therapy is challenging, since at this stage, there is no clear consensus on the best material or encapsulated agents to use.

### 11.2 Applications in Experimental ICH

To date, preclinical ICH studies testing hydrogels have used the collagenase, autologous blood, or FeCl_2_ models (Table 2). Although no animal model can fully recapitulate the complex etiology of human ICH, each model can provide important information on the pathophysiological processes that could be therapeutically targeted. When designing a study, it is important to consider how model selection could influence the outcomes of hydrogel injection. For example, the collagenase model causes more severe BBB disruption, cell death, and peripheral immune cell infiltration than the autologous blood model,^[^ ^94^ ^]^ which may influence the inflammatory response to the material and how quickly the hydrogel is degraded. MacLellan et al. also reported quicker resolution of neurological deficits in the autologous blood model, suggesting that studies investigating long‐term outcomes should consider using the collagenase model.^[^ ^94^ ^]^ Nonetheless, both models produce consistent hemorrhages with pathophysiological elements observed in human ICH, with each model having advantages depending on the research question to be answered. The FeCl_2_ model mimics iron‐induced toxicity and the resultant edema after ICH,^[^ ^95^ ^]^ but does not model the rise in ICP caused by hematoma mass effects and thus may be an inappropriate model for optimizing the time and volume of hydrogel administration.

While a number of hydrogel candidates tested in IS and TBI are now being explored in ICH, the unique environment of the ICH cavity, including the presence of free iron and peripheral immune cell populations, may differentially affect the therapeutic efficacy of injected hydrogels. In addition, the majority of ICH models result in striatal lesions, whereas TBI models predominantly trigger injury to the cortex^[^ ^96^ ^]^ and IS models can cause damage to both subcortical and cortical areas, depending on the severity and location of the arterial occlusion.^[^ ^97^ ^]^ As a result of differences in injury proximity to the neurogenic zones, the type of neurological deficit caused, and the tissue architecture of the affected region, therapeutic strategies employed in one type of acute brain injury may not necessarily be translatable across disease models.

When considering clinical translation for ICH, the proven safety of minimally invasive hematoma aspiration could present an opportunity for hydrogel transplantation.^[^ ^29^ ^]^ Indeed, the Hemorrhagic Stroke Academia Industry (HEADS) report outlining recommendations and priorities for future translational ICH research encouraged further study of “multimodal therapy” such as clot evacuation combined with administration of a restorative therapy.^[^ ^98^ ^]^ However, as discussed, administration of hydrogels or other therapeutic agents must be carefully timed alongside ICH progression, to maximize the safety and efficacy of the therapy. Alternatively, hydrogel administration at chronic timepoints may be safe without the need for hematoma evacuation. Moreover, the neutral outcome of early minimally invasive surgery and the unsuccessful trials of multiple interventions aimed at reducing primary injury cast doubt on the early intervention or “neuroprotective” approach in ICH.^[^ ^28^ ^]^ Studies exploring hydrogel administration at chronic timepoints in combination with pro‐regenerative therapies are therefore a potential alternative approach.

## 12 Conclusions and Future Perspectives

Although there are differences in the pathophysiological features of IS, TBI, and ICH, some mechanisms are shared, including brain tissue repair processes. Given these commonalities, the extensive literature in IS and TBI can inform and advance the newly emerging field of hydrogel use in ICH. The wide variety of hydrogels tested in IS and TBI models gives scope to repurpose these materials for ICH applications, perhaps with ICH‐specific therapeutics for a combinatorial approach. Additionally, cell therapies administered with hydrogels have been extensively explored in IS and TBI and may also be worth considering in ICH. Studies in IS and TBI have also revealed some beneficial effects of hydrogel implantation on endogenous repair processes, and therefore assessment of angiogenic and neurogenic responses following hydrogel administration may be valuable in the context of ICH.

Conversely, the unique features of ICH pathology may preclude direct translation of methods from IS and TBI research and require a more tailored approach. In particular, the volume and timing of hydrogel administration must be carefully considered, given the space‐occupying nature of the ICH lesion. Furthermore, several aspects of hydrogel implantation such as in vivo hydrogel degradation and long‐term local and systemic effects are yet to be extensively explored in acute brain injury, and therefore require further study before clinical translation. Finally, the proven clinical safety of surgical intervention after ICH may represent an exciting opportunity in terms of the feasibility and translational potential of hydrogel‐based therapeutics.

## Conflict of Interest

The authors declare no conflict of interest.

## Author Contributions

J.M.T., I.L., F.B., and O.‐R.S. contributed equally to this work. J.M.T., I.L., F.B., and O.‐R.S. were responsible for the concept of the review, literature search, and writing of the paper. S.M.A., C.B.L., and E.P. contributed to critical review of the paper. All authors read and approved the final paper.
